# Differential Rapid Plasticity in Auditory and Visual Responses in the Primarily Multisensory Orbitofrontal Cortex

**DOI:** 10.1523/ENEURO.0061-20.2020

**Published:** 2020-06-02

**Authors:** Sudha Sharma, Sharba Bandyopadhyay

**Affiliations:** 1 Advanced Technology Development Centre, Indian Institute of Technology Kharagpur, WB-721302, India; 2 Department of Electronics and Electrical Communication Engineering, IIT Kharagpur, WB-721302, India

**Keywords:** multisensory, oddball, orbitofrontal cortex, rapid plasticity, stimulus value, visual bias

## Abstract

Given the connectivity of orbitofrontal cortex (OFC) with the sensory areas and areas involved in goal execution, it is likely that OFC, along with its function in reward processing, also has a role to play in perception-based multisensory decision-making. To understand mechanisms involved in multisensory decision-making, it is important to first know the encoding of different sensory stimuli in single neurons of the mouse OFC. Ruling out effects of behavioral state, memory, and others, we studied the anesthetized mouse OFC responses to auditory, visual, and audiovisual/multisensory stimuli, multisensory associations and sensory-driven input organization to the OFC. Almost all, OFC single neurons were found to be multisensory in nature, with sublinear to supralinear integration of the component unisensory stimuli. With a novel multisensory oddball stimulus set, we show that the OFC receives both unisensory as well as multisensory inputs, further corroborated by retrograde tracers showing labeling in secondary auditory and visual cortices, which we find to also have similar multisensory integration and responses. With long audiovisual pairing/association, we show rapid plasticity in OFC single neurons, with a strong visual bias, leading to a strong depression of auditory responses and effective enhancement of visual responses. Such rapid multisensory association driven plasticity is absent in the auditory and visual cortices, suggesting its emergence in the OFC. Based on the above results, we propose a hypothetical local circuit model in the OFC that integrates auditory and visual information which participates in computing stimulus value in dynamic multisensory environments.

## Significance Statement

Understanding encoding of different modalities in single neurons of orbitofrontal cortex (OFC) can help decipher its role in perceptual decision-making. Basic sensory response properties of mouse OFC are poorly understood. In our study mouse OFC is found to be primarily multisensory with varied nonlinear interactions, explained by parallel unisensory and multisensory inputs. Audiovisual associations cause rapid plastic changes in the OFC, which bias visual responses while suppressing auditory responses. Similar plasticity was absent in the sensory cortex. Thus, the observed intrinsic visual bias in the OFC weighs visual stimuli more than associated auditory stimuli in value encoding in a dynamic multisensory environment.

## Introduction

The neurophysiological properties of the orbitofrontal cortex (OFC) along with its connections with the sensory and limbic structures place the OFC in a unique position (Carmichael and Price, 1995; Rolls, 2004), where it can potentially use different sensory inputs to integrate and modulate them to perform value computation and constant updating based on dynamic needs. This central position of OFC also implicates it for its likely role in perception-based decision-making, with no reward value attached. Although studies of the function of the OFC have mainly used unisensory stimuli (Sadacca et al., 2018), the real-world sensory stimuli are mostly multisensory, either modulating the perception of each other or are associated with each other. Thus, to understand the function of the OFC in perception-based multisensory decision-making, it is important to consider the circuits involved in providing multisensory information to the OFC, representation of multisensory stimuli in the OFC and how the representations may change with associations, purely from a sensory point of view. Such understanding provides the crucial background representation of the sensory world on top of which, reward-punishment (O'Doherty et al., 2001), action-outcome (Simon et al., 2015), and prediction error (Schultz, 2016) signals operate to achieve goal-directed behavior in a seamless manner. Further, the outputs of the OFC based on such multisensory information can potentially also modulate representation in lower order primary sensory regions (Winkowski et al., 2013) to aid behavior.

There have been numerous studies about audiovisual integration such as encoding of vocalizations and effect of simultaneous presentation of face stimuli in nonhuman primates (Sugihara et al., 2006). Studies on superior colliculus of cats and nonhuman primates forms the bulk of literature in the field of multisensory processing (Cuppini, 2010). Our understanding of multisensory processing is yet limited primarily because of the dearth of tools available to study specific circuits in animal models other than mice. Prefrontal cortex in mice still lacks proper definition which makes it difficult to compare regions across species. Hence it becomes important that findings from nonhuman primates be understood and characterized in mice (Carlén, 2017). Lower order organisms such as *Drosophila* have provided an insight into the mechanism being a multilevel multimodal convergent process (Ohyama et al., 2015), which may also be the case for comparatively higher order organisms. Thus, using a mouse as the animal model for studying multisensory processes, optogenetic tools can help us decipher the specific circuits involved which will greatly benefit our understanding about multisensory processing. Therefore, a detailed anatomic and physiological study of the mouse OFC in encoding different sensory inputs and integrating them in its unique way to extract the best possible estimate of the environment needs to be undertaken.

In this article, using auditory and visual stimuli, we study the underlying multisensory representation and multisensory response properties in the mouse OFC. With retrograde labeling studies, we show the possible origins (direct and indirect) of auditory and visual inputs to the OFC. The origins of multisensory inputs lie in nonprimary auditory and visual cortices which we show have multisensory responses as well. Convergence of sensory signals in OFC is known from work of Edmond Rolls only for nonhuman primates and not mice. Using a novel multisensory oddball paradigm, we show that the OFC receives exclusive unisensory (auditory and visual) as well as exclusively multisensory inputs. Further, here we distinctly show that both unisensory and multisensory (audiovisual) signals parallelly converge to the OFC, which was not known, even in primates. Finally, we show that long auditory-visual associations induce rapid plasticity in the OFC, which is differential in nature, showing strong suppression to auditory responses with an effective enhancement of visual responses. Such association driven rapid plasticity is absent in the sensory cortices, which provide input to the OFC and could thus be emergent in the OFC. The observed visual bias in the OFC due to multisensory associations has a number of implications in understanding goal-directed flexible behavior in a realistic multisensory environment and in understanding neuropsychiatric disorders associated with OFC dysfunction.

## Materials and Methods

All procedures were approved by the Indian Institute of Technology, Institutional Animal Care and Use Committee. Animals were maintained in 12/12 h light/dark cycle, and all experiments were performed during the dark cycle on the C57 strain from The Jackson Laboratory.

### Anatomy

Mice aged more than postnatal day (P)60 (>P60, male or female) were anesthetized with isoflurane (5% induction and 1.5% maintenance) and placed on a stereotaxic frame. The body temperature was kept at 37°C throughout the procedure using a heating pad. An incision was made to expose the skull. A burr hole (∼0.5 mm in diameter) was made above the injection site. Tracers were loaded in a glass micropipette mounted on a Nanoject II attached to a micromanipulator and then injected at a speed of 20 nl/min. Retrobead injections were targeted stereotactically into the left OFC using the following coordinates (Paxinos and Franklin, 2013): +2.5 mm from the bregma point and 1 from the midline at a depth of 1.8 mm. After recovery from anesthesia, mice were left in their home cages for two weeks following the injection.

For histology and quantification, mice were deeply anesthetized with isoflurane, perfused transcardially with 0.1 m PBS followed by 4% paraformaldehyde (PFA). Brains were harvested and after a postfixation period of 8–10 h in 4°C, 100-μm-thick sections were cut using a vibratome (Leica VT1000S), and images were taken in a fluorescence microscope (Leica DM2500). Brain slices encompassing the span of auditory cortex (ACX) rostro-caudally were selected, areas were demarcated based on a mouse brain atlas (Paxinos and Franklin, 2013), and labeled cell bodies were counted manually in each of the different regions on all sections.

### Electrophysiological recordings

**Figure 1.**
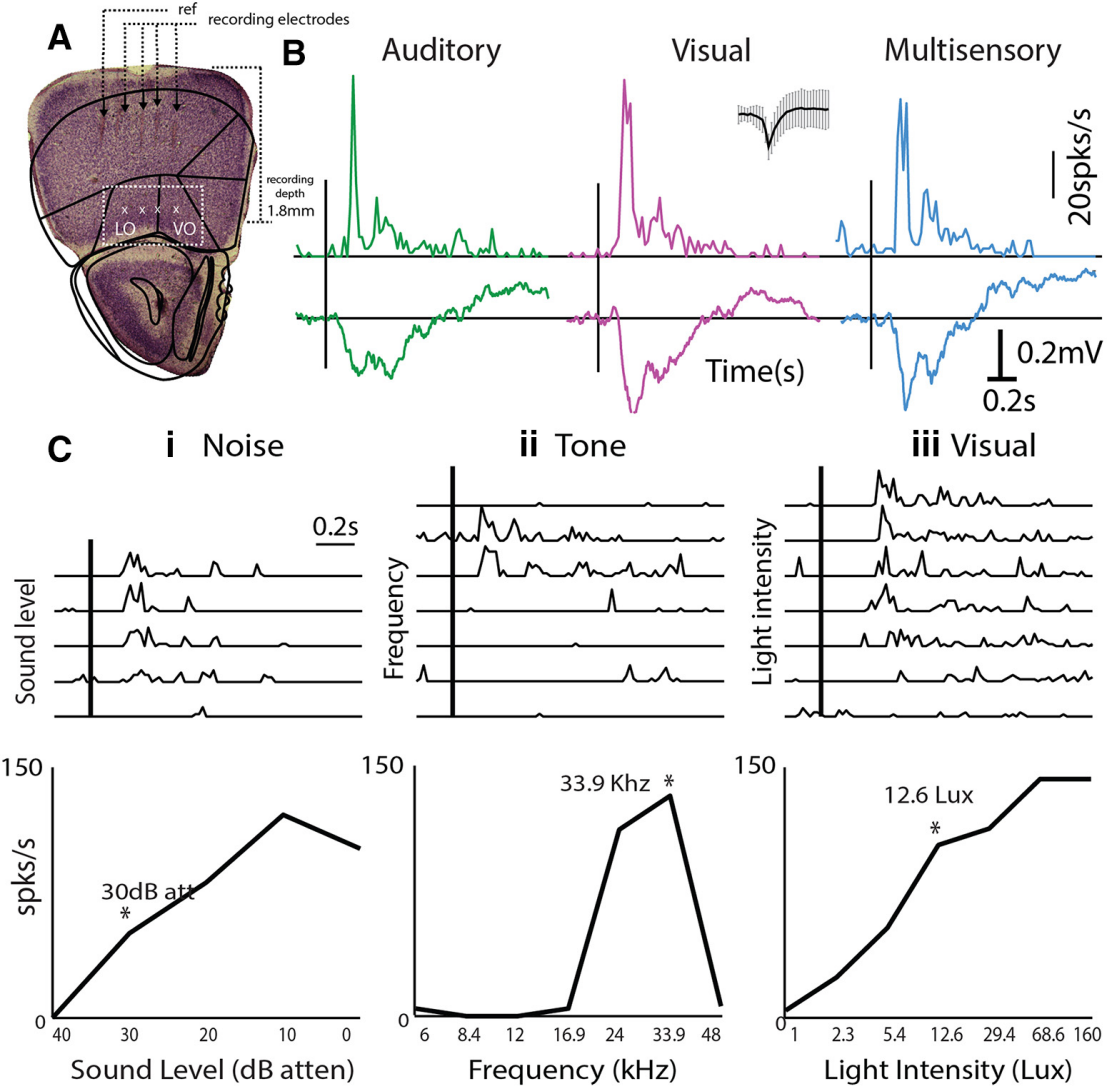
Multisensory responses of mouse OFC single neurons. ***A***, left, Example of electrode tracks (arrowheads) shown in Nissl stains of brain slices showing recording locations in Lateral OFC/Ventral OFC (white box). ***B***, Representative PSTHs of a single OFC neuron in response to auditory (tone = 33.9 kHz, green), visual (LED blink 12.6 lux, pink), and multisensory stimuli (blue). Inset, Spike shape. Mean LFPs on the same channel are shown below each PSTH. ***Ci–iii***, top row, PSTHs of single neurons in response to multiple sound levels of a noise burst (***i***), multiple frequencies (***ii***), and multiple intensities of LED blink (***iii***) are shown. Bottom row, The mean rate response as a function of sound level, frequency, and light intensity are plotted. Asterisks represent the chosen values presented for the *M* stimulus in each case.

Mice (58 mice P31–P40 and two mice P27 and P28) were anesthetized with isoflurane (5% induction and 1% maintenance) and the skull was attached to a stainless-steel plate for fixing the head of the animal for inserting the electrodes and performing recordings. Animals were kept warm throughout the experiment by maintaining an external body temperature of 37°C throughout the procedure using a heating pad. An incision was made to expose the skull. A craniotomy (∼2 mm in diameter) was made above the left OFC (or ACX) for recordings (+2.5 mm from bregma and +1 mm from the midline, coordinates of ACX; Winkowski et al., 2013). Extracellular recordings were performed using tungsten microelectrodes array (MEA) of impedance 3–5 MΩ (MicroProbes); 4 × 4 custom-designed metal MEAs with an interelectrode spacing of 125 μm were used. The array was advanced slowly to a depth of 1800 μm from the surface (Fig. 1*A*) into the OFC using a micromanipulator (MP-285, Sutter Instrument Company). The electrodes were allowed to settle for >20 min before the stimulus presentation was started. Signals were acquired after passing through a unity gain head stage (Plexon, HST16o25) followed by PBX3 (Plexon) preamp with gain of 1000×, to obtain the wideband signal [used to extract local field potential (LFP), 0.7 Hz to 6 kHz] and spike signals (150 Hz to 8 kHz) in parallel and acquired through National Instruments Data Acquisition Card (NI-PCI-6259) at 20-kHz sampling rate, controlled through custom-written MATLAB (MathWorks) routines. Further, all online/offline analysis was performed using custom-written MATLAB routines. After the experiment was completed, the brain of the animal was routinely isolated and kept in a 4% solution of PFA for later visualization of the site of recording in 100-μm-thick sections through cresyl violet staining.

### Auditory and visual stimulation

Sound stimuli were presented to the ear contralateral to the recording hemisphere inside a soundproof chamber (IAC Acoustics), 10 cm away from the right ear (contralateral) of the mouse, with TDT electrostatic speakers (ES1) driven by TDT drivers ED1 after attenuation by TDT attenuators (PA5), generated through TDT RX6 using custom software written in MATLAB. The acoustic calibrations, performed with microphone 4939 (Brüel&Kjær), of the ES1 speakers (TDT) in the sound chamber, showed a typical flat (±7 dB) calibration curve from 4 to 60 kHz; 0-dB attenuation on PA5 corresponded to a mean (across frequency) of 95-dB SPL. Sound stimuli consisted of pure tone (6–48 kHz) or white noise (bandwidth 6–48 kHz) bursts. After usual initial characterization of single units with different intensity noise bursts (five repetitions, 50-ms duration, 5-ms rise and fall), single-unit responses to tones of different frequencies (five repetitions each, 6–48 kHz, ½ octaves apart, 50-ms duration with 5-ms rise and fall, 5-s interstimulus interval at ∼65- to 75-dB SPL, usually ∼10–20 dB louder than the usual noise threshold) were collected. A particular frequency was decided based on the maximum number of responsive channels to the different tones for additional experiments and for audiovisual stimulation (stimulus *A*). Single-unit spike times were obtained from the acquired spike channel data using threshold crossing and spike sorting with custom-written software in MATLAB. Responses to *A* with 30 repetitions were collected following the characterization and used for further analysis.

For visual stimulation, a white LED [5-mm Round White LED (T-1 3/4)] kept 5 cm away from the eye of the animal was used. Full-field illumination to the contralateral eye was provided and the intensity was varied through the NI DAQ card, using MATLAB routines. LED blink stimulus was presented for 10 ms (*V* stimulus) with an interstimulus interval of 5 s in order for the cones in the retina to recover. LED flash of 10-ms duration allowed for response rates of the *A* and *V* to be comparable. A petroleum jelly-based eye ointment was applied to prevent the eye from drying. Single-unit rate responses were obtained for varying intensities of the LED (1–160 lux, five to seven steps, Model: LX1010b, Digital Lux Meter, 0–20,000 lux), and threshold intensity was determined. For additional experiments and audiovisual stimulation, a chosen intensity above the threshold (of stimulus *V*) was used, which corresponded to 5–30 lux. As with the *A* stimulus, responses to *V* with 30 repetitions were collected.

Bimodal stimulation and audiovisual pairing comprised of simultaneous presentation of the *A* and *V* stimuli (*M*), as above; 100 repetitions of the *M* stimuli were presented and the first 30 were considered for calculating the *M* response for comparison with responses to unisensory stimuli. Following the 100 presentations of the *M* stimulus, considered as the *A* and *V* pairing, 30 repetitions of *A* and then *V* stimuli were presented to obtain the effect of audiovisual pairing. For comparisons between before and after pairing, the two mice aged <31 d were not included due to possible immaturity in the visual system. Further, for the pairing dataset only, units with the same spike shapes present in all five sets of responses (*A*-before, *V*-before, *M*, *A*-after, and *V*-after) were included. Thus, of the total 158 units obtained in 12 animals with responses to *A*, *V*, and *M*, 91 units were used for the analysis of the effect of pairing.

### Data analysis

Spike sorting was done offline in custom-written MATLAB scripts. Data were baseline corrected and notch filtered (Butterworth fourth order) to reject any remnant power supply 50-Hz oscillations. Wideband data were bandpass filtered for LFPs between 1 and 500 Hz, and spiking activity was obtained directly from the spike channels of the PBX3 preamp. Deviations above 4 SDs from the baseline were isolated, and based on shapes, spike waveforms were clustered into different groups.

#### Calculation of latencies, spike rate, peristimulus time histogram (PSTH), and response duration

OFC and ACX have different time scales of response to the different stimuli. For the ACX response characterization, 5-ms binning was done for calculating spike rates. For OFC, we found that the same bin size as ACX did not capture the response dynamics, due to larger spike jitter in OFC across trials, leading to noisier PSTHs. Thus, we used different bin sizes for analyzing response from OFC and ACX. These choices however do not change any result fundamentally. For data from OFC, a moving window of size 100 ms in steps of 20 ms (ACX, 50-ms window in steps of 5 ms) after the stimulus start was used for comparison with a random 100-ms window from the baseline (between 400 and 10 ms preceding stimulus onset) for detecting a significant response (*p *<* *0.05, paired *t* test). The middle of the first significant window was taken as the latency of response. The time bin (20-ms bins for OFC, 5 ms for ACX) corresponding to the maximum spike rate within 500 ms of stimulus onset was detected. Mean spike rate in a 100-ms window (ACX: 25 ms for *A* and *V*, 50 ms for *M*) around the peak was taken as the spike rate. All PSTHs shown were calculated based on 20-ms bins (5-ms bins for ACX) by averaging the firing rate from multiple repetitions of the same stimulus. A sliding response window of size 100 ms (50 ms for ACX), starting from the stimulus start, in 20-ms steps (5 ms for ACX), was compared with the random 100-ms window in the baseline for significance. Consecutive significant bins with a time difference of <100 ms (25 ms for ACX) between were joined together in the response for the determination of response duration. The LFP signal was converted to root mean square (rms) in the same time windows used for spike data and significance was calculated in a similar manner as above. The same procedure was followed for both OFC and ACX data as described above for the case of spikes. The LFP response strength was calculated as the rms of the average LFP waveform in a 100-ms window centered around the maximum dip in LFP following stimulus onset.

#### Classification of neurons

Neurons that responded both to auditory and visual stimulation or showed significant change on presentation of multisensory stimulus when compared with the larger of the unisensory responses are considered as multisensory units. Neurons that were responsive to only auditory or visual and do not show any significant change on multisensory stimulus presentation are considered as exclusively auditory or exclusively visual units respectively. To calculate percent change on multisensory presentation, multisensory enhancement index (MEI) = [*r*(*M*) – *max*(*r*(*A*),*r*(*V*)]/*max*(*r*(*A*),*r*(*V*)) is used, where *r*(*X*) is considered as the rate response to stimulus *X *=* A*, *V*, or *M*. For comparison of spike rates to the auditory and visual stimulus, unisensory imbalance (UI) is calculated as (*r*(*V*) – *r*(A))/(*r*(*V*) + *r*(A)) (Miller et al., 2015).

#### Comparisons of slopes

First a straight line through the origin (*y* = *mx*) was fit using minimum mean squared error, to the scatter data of after versus before responses and the slope was estimated. For comparisons of slopes of the best fit lines to scatter plots of after pairing responses versus before pairing responses across conditions, bootstrap analysis (Efron and Tibshirani, 1993) was performed to obtain confidence intervals on the slopes. Bootstrap populations (*n *=* *1000) were created by randomly sampling before-after pairs of responses with replacement from the original dataset. We obtained 1000 slope values from the population of 1000 bootstrap sets. The slopes from two different conditions were considered to be significantly different if the 95% confidence intervals of the two did not overlap with each other and accordingly for one-sided comparisons.

## Results

### Single neurons in the mouse OFC are primarily multisensory

In order to investigate the relative prevalence of multisensory neurons and interaction of two unisensory stimuli in the mouse OFC, we first characterize responses of single neurons to an auditory, a visual and a multisensory stimulus (Fig. 1*B*). Single-unit recordings (Materials and Methods) were performed in the anesthetized mouse OFC. Recording site depth and the location were determined *post hoc* by cresyl violet staining of the coronal sections of the mouse brain for all recorded animals (Fig. 1*A*, example). Usually, intensity of presentation above noise threshold was first determined based on responses to broadband noise at different intensities (Fig. 1*Ci*, asterisk) and then tuning of single units was obtained with tone presentations (Fig. 1*Cii*; Materials and Methods) at 65- to 75-dB SPL. A particular frequency (Fig. 1*Cii*, asterisk) was chosen for the auditory component (*A*) of the multisensory stimulus (*M*). The intensity of the visual component (*V*) of the subsequent multisensory stimulus was determined based on responses of single units to the visual stimulus at multiple intensities (Fig. 1*Ciii*, asterisk; Materials and Methods). Overall, data from 158 (80% of all recorded units) single units in the mouse OFC across 12 animals were analyzed for unisensory and multisensory response characterization. These units showed a significant response to at least one of the three stimuli, *A*, *V*, and *M*.

Most neurons (96.2% of responding neurons, *n *= 152/158) in the OFC were found to be multisensory in nature; 147 units responding to auditory as well as visual stimuli and five units did not respond to both *A* and *V* but were multisensory in nature (two responded to only *A*, and two to only *V* and suppressed to *M* and one unit responded to neither *A* nor *V* but responded to *M*). Representative response PSTHs (20-ms bin) to each of the three stimuli *A*, *V*, and *M*, for a multisensory neuron, are shown in Figure 1*B*. LFPs recorded simultaneously from the same electrode are shown below the PSTHs, which also show similar characteristics. The remaining six units were found to be exclusively auditory (same response to *M* as to *A*) and none were exclusively visual. Similar to early sensory cortices, neurons in the OFC were also frequency selective (Fig. 1*Cii*), and these neurons were found to be sensitive to increases in the intensity of the auditory and visual stimuli (Fig. 1*Ci*,*iii*). Intensities of *A* and *V* chosen for the presentation of *M* were within the general dynamic range of the neurons and not at saturating activity levels.

**Figure 2.**
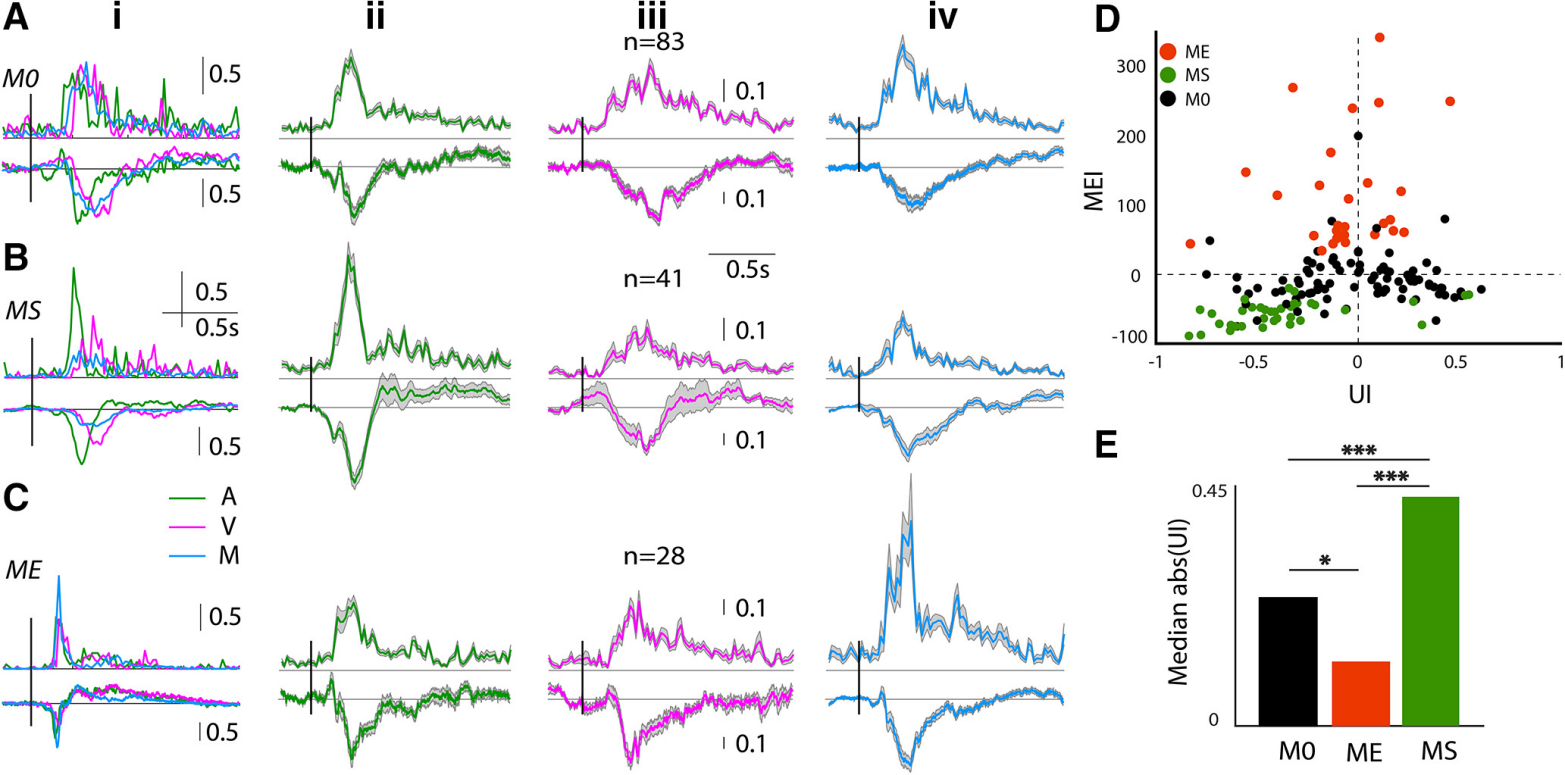
Multisensory modulation of mouse OFC single neurons. ***A–C***, Each row shows representative examples of single-unit PSTHs (left column) in response to *A* (green), *V* (pink), and *M* (blue) followed by population PSTHs with SEM (gray shading; normalized by the maximum response of the *A* and *V* stimuli) in response to *A* (second column), *V* (third), and *M* (fourth). ***A***, No modulation (*M0*). ***B***, Suppressed (*MS*). ***C***, Enhanced (*ME*). ***D***, Scatter plot of MEI versus UI, each dot represents a single unit and shade indicates the kind of modulation. ***E***, Barplots showing median absolute (UI) of the groups of three kinds of modulation (*p < 0.05, ***p < 0.001); *n* represents the number of single neurons, vertical bars over the example neurons and population PSTHs is the length of normalized rate responses (a.u.).

We further characterize the multisensory responses of OFC single units based on the modulation of unisensory responses because of the simultaneous presence of a stimulus of another modality, in the multisensory stimulation. Almost equal proportions of neurons showed significant modulation by *M* (45.3% 69/152; Fig. 2) and no modulation by *M* (54.3%, 83/152). The proportions of the two types of neurons were not significantly different (two-proportion *z* test, *z* = −1.60, *p* = 0.1074). The neurons with no modulation by *M* responded to both *A* and *V* but their rate responses to *M* were not significantly different from the larger of the responses to the unisensory stimuli (example neuron; Fig. 2*Ai*). Thus, although the response rates to each of the unisensory stimuli (*A* and *V*) were individually not saturating, presentation of the two stimuli together did not significantly alter the response strengths. Thus, these neurons effectively show nonlinear interactions between the two stimuli when presented simultaneously. Out of the remaining 45.3% neurons that significantly altered their spike rates on presentation of *M* compared with the unisensory conditions, a significantly larger proportion of neurons (41 units, 26.9%, *p* < 0.01, binomial test) showed suppression of responses (example neuron; Fig. 2*Bi*) compared with enhancement (28 units, 18.3%, example neuron; Fig. 2*Ci*). The corresponding LFPs (shown below each PSTH) collected on the same channels as the units show similar character in terms of enhancement and suppression (Fig. 2*B*,*C*, bottom row). Population PSTHs of each set of neurons, no modulation (*M0*), suppression (*MS*), and enhancement (*ME*) are shown in Figure 2*A–C*, along with population mean LFPs, in response to the three stimuli (columns *ii–iv*) below the population PSTHs. To quantify the degree of modulation, the MEI (Materials and Methods) was calculated. The scatter plot of MEI of each unit versus the UI (Materials and Methods) between the rate responses to *A* and *V* (Fig. 2*D*) show that most suppressed units had a high absolute UI, while enhanced units had lower UI. The median absolute (UI) of the three classes of multisensory modulation were significantly different from each other [Kruskal–Wallis with *post hoc* Tukey’s HSD, χ^2^(df = 151) = 36.89, *p* < 10^−9^, *MS* and *ME* (*p* = 0.0), *M0* and *ME* (*p* = 0.07), *M0* and *MS* (*p* = 0.0)] with medians, *M0* = 0.24, *MS* = 0.43, *ME* = 0.12 (Fig. 2*E*). Thus, UI appears to be a determining factor of whether a neuron in the OFC would be suppressed or enhanced or show no changes. The median absolute (UI) values for units with larger auditory (LA) responses (negative UI) was higher (Wilcoxon rank-sum test, *z* = 2.18, *p* = 0.02) than units with larger visual (LV) responses, indicating OFC neurons at the chosen light intensity of *V* and frequency and sound level of *A* had stronger responses to *A* than *V*. However, the distribution of observed absolute (UI) for LA and LV units were not different (two-sample Kolmogorov–Smirnov test, *p* = 0.11). The above indicates that the suppression of units is due to having LA responses which coincide with higher UI in this dataset. Units with high positive UI (LV) do not get suppressed, while larger fraction (two-proportion *z* test, *z* = 7.5, *p* = 10^−5^) of LA units gets suppressed. Thus, the primary effect of the presentation of *M* is to inhibit the responses of neurons responding strongly to *A*.

### Response latency and response duration indicate modality-specific coding

**Figure 3.**
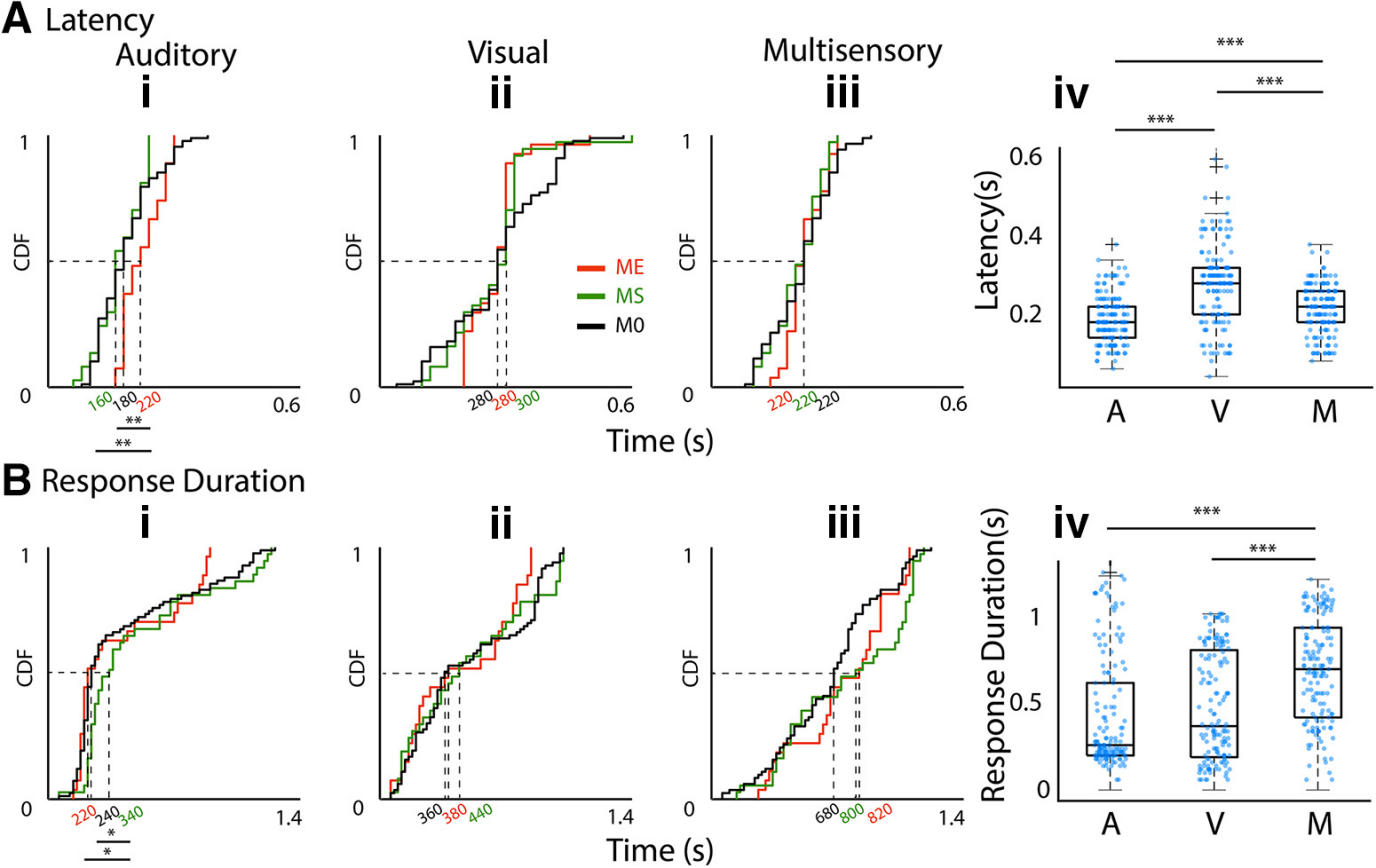
Dependence of latency and response durations of OFC units to *A*, *V*, *M* stimuli. ***Ai–iii***, CDF plots of latencies of OFC single units with different types of modulations (identified by shading of lines) to *A*, *V*, and *M* (***i–iii***) stimuli. ***Aiv***, Box plots of latencies of all single units to each of the stimuli, *A*, *V*, and *M*. ***Bi–iv***, Same arrangement of CDF plots and box plots as in ***A*** for response duration. ***p < 0.001.

As the mean firing rate response of OFC single neurons to each of the unisensory modalities (*A* and *V*) determine multisensory modulation we also investigate how their basic temporal response properties relate to the type of modulation. Figure 3*Ai–iii* shows the cumulative response distribution functions (CDFs) of response latency of all neurons to the three stimuli *A*, *V*, and *M* (*i–iii*) separated into groups by their modulation index (identified by colors). Comparing the median latency between the three stimulation conditions (*A*: 180 ms, *V*: 280 ms, and *M*: 220 ms; Fig. 3*Aiv*) shows that responses to *A* have significantly lower latency than the other two cases [Kruskal–Wallis with *post hoc* Tukey’s HSD, χ^2^(df = 455) = 79.36, *p* < 10^−18^, *A* and *V* (*p* = 0.0), *A* and *M* (*p* = 0.006), *V* and *M* (*p* = 0.0)], while responses to *V* have significantly higher latency than that of *M*. The response latencies to *V* and *M* were not different in the *ME*/*MS*/*M0* types of units (Kruskal–Wallis with *post hoc* Tukey’s HSD, for *V*, χ^2^(df = 151) = 0.28, *p* = 0.86, for *M*, χ^2^(df = 151) = 0.9, *p* = 0.63). However, *ME* units had significantly longer response latency to *A* than *MS* and *M0* units [Kruskal–Wallis with *post hoc* Tukey’s HSD, χ^2^(df = 151) = 12.74, *p* = 0.0017, *ME* and *MS* (*p* = 0.002), *ME* and *M0* (*p* = 0.003)]. Thus, long latency *A* responses, indicative of weaker auditory drive, were usually enhanced as also concluded from rates above. Thus, these observations are further evidence that the strength of auditory drive in OFC units determines the kind of modulation the units have with a multisensory stimulus.

Unlike in the primary auditory and visual sensory cortices, *A*, *V*, and *M* stimuli evoked a considerably long-duration response and LFPs (Figs. 1*B*, 2) in the OFC. Response duration CDFs for each stimulus separated into groups based on their multisensory modulation (Fig. 3*Bi–iii*) show that in case of the *V* stimulus-response durations were independent of multisensory modulation (Kruskal–Wallis with *post hoc* Tukey’s HSD, χ^2^(df = 146) = 0.86, *p* = 0.65). However, median *A* response duration was significantly shorter for *ME* (220 ms) units than that of *MS* (340 ms, Wilcoxon rank-sum test, *z*(df = 62) = −1.96, *p* < 0.04), which is significantly longer than that of M0 (240 ms, Wilcoxon rank-sum test, *z*(df = 118) = 2.35 *p* = 0.01). Thus, again, weaker auditory drive, indicated by short duration responses, coincides with multisensory enhancement while stronger auditory drive, indicated by long response duration, coincides with multisensory suppression. The median response duration of OFC single units, to *A*, *V*, and *M* stimuli (Fig. 3*Biv*), shows that the *M* stimulus (720 ms) evoked significantly longer responses compared with the *A* (260 ms, *p* = 0.0) and *V* (380 ms, *p* = 0.0; Kruskal–Wallis with *post hoc* Tukey’s HSD) stimuli, possibly indicating temporal summation with the simultaneous presentation of two stimuli.

Thus, based on short response latency and long response duration, it is further corroborated that the higher strength of auditory input, as also determined by rates, indicated multisensory suppression. Weak auditory drive indicated by long latency and short duration responses indicated multisensory enhancement. Multisensory modulation is largely independent of the response to the unisensory visual component of the multisensory stimulus.

Our results based on response rates and basic temporal response characteristics suggests stimulus type (*A*, *V*, *M*)-specific coding as each of the stimuli have different median latencies of response, different response strengths to *A* and *V*, and longer duration responses to *M* compared with *A* and *V*. Further, simultaneous presentation of *A* and *V* leads to variety of nonlinear interactions between the individual unisensory responses. All the above, especially different response latencies to *A*, *V*, and *M* suggest that each of the three types of stimuli activates partially unique and partially overlapping pathways. The possible mechanism which may lead to such effects is described with a hypothetical network model later in the paper.

### Sources of auditory, visual, and multisensory inputs to the mouse OFC

**Figure 4.**
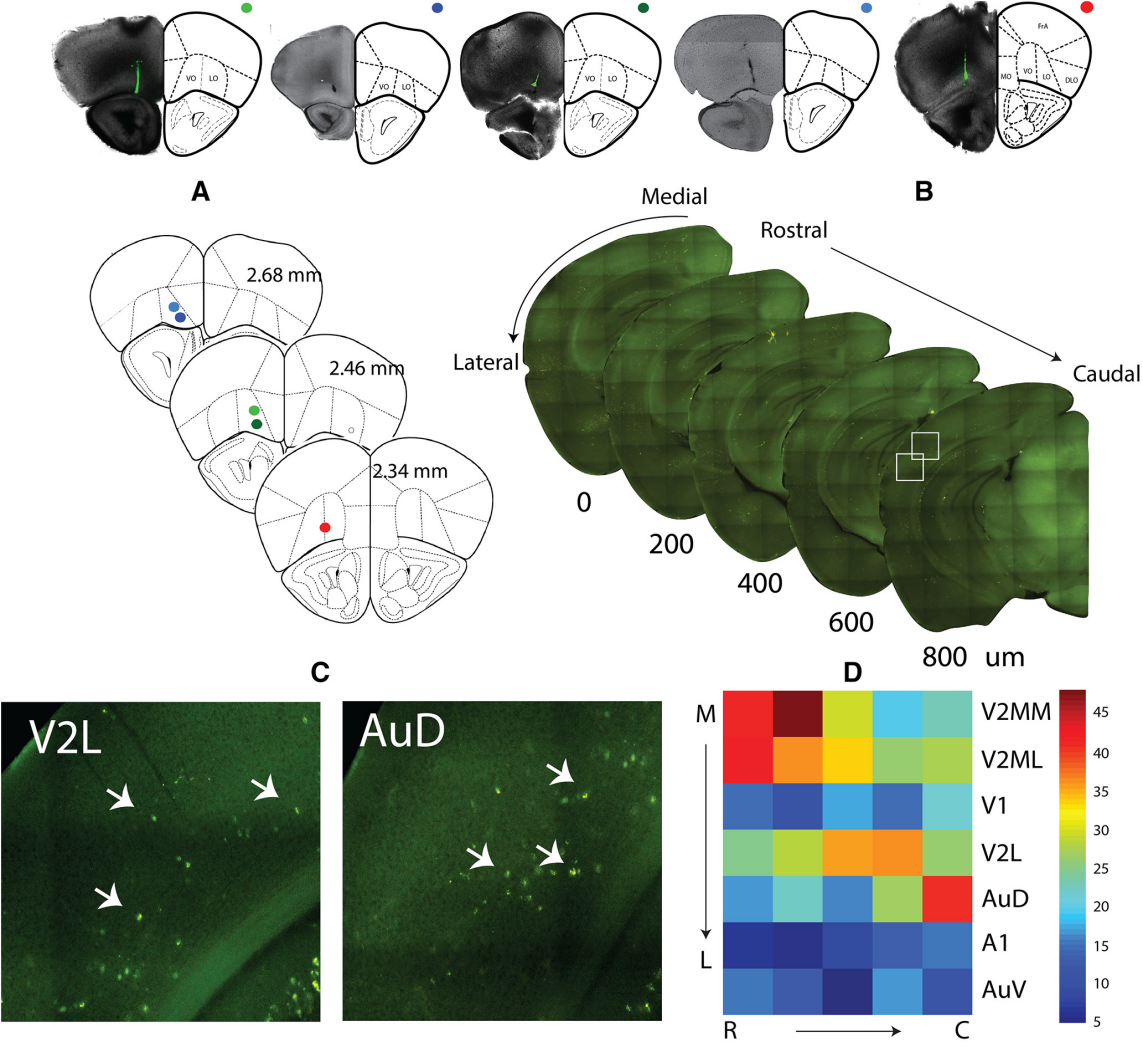
Origin of OFC afferents in the early sensory cortex, carrying auditory, visual, and multisensory information. Top row, Brightfield images of the frontal cortex section with retro bead injection site in OFC. Frontal cortical regions overlaid from mouse atlas, ***A***, Summary data of injection sites from five animals in the top row. ***B***, Example sequence of alternate 100-μm fluorescent images of sections encompassing rostrocaudal extent of the ACX. ***C***, Sample areas in AuD and V2L (white boxes in ***B***) with retro bead labeled cell bodies (marked by arrows). ***D***, Number of labeled cells observed in the different areas.

**Figure 5.**
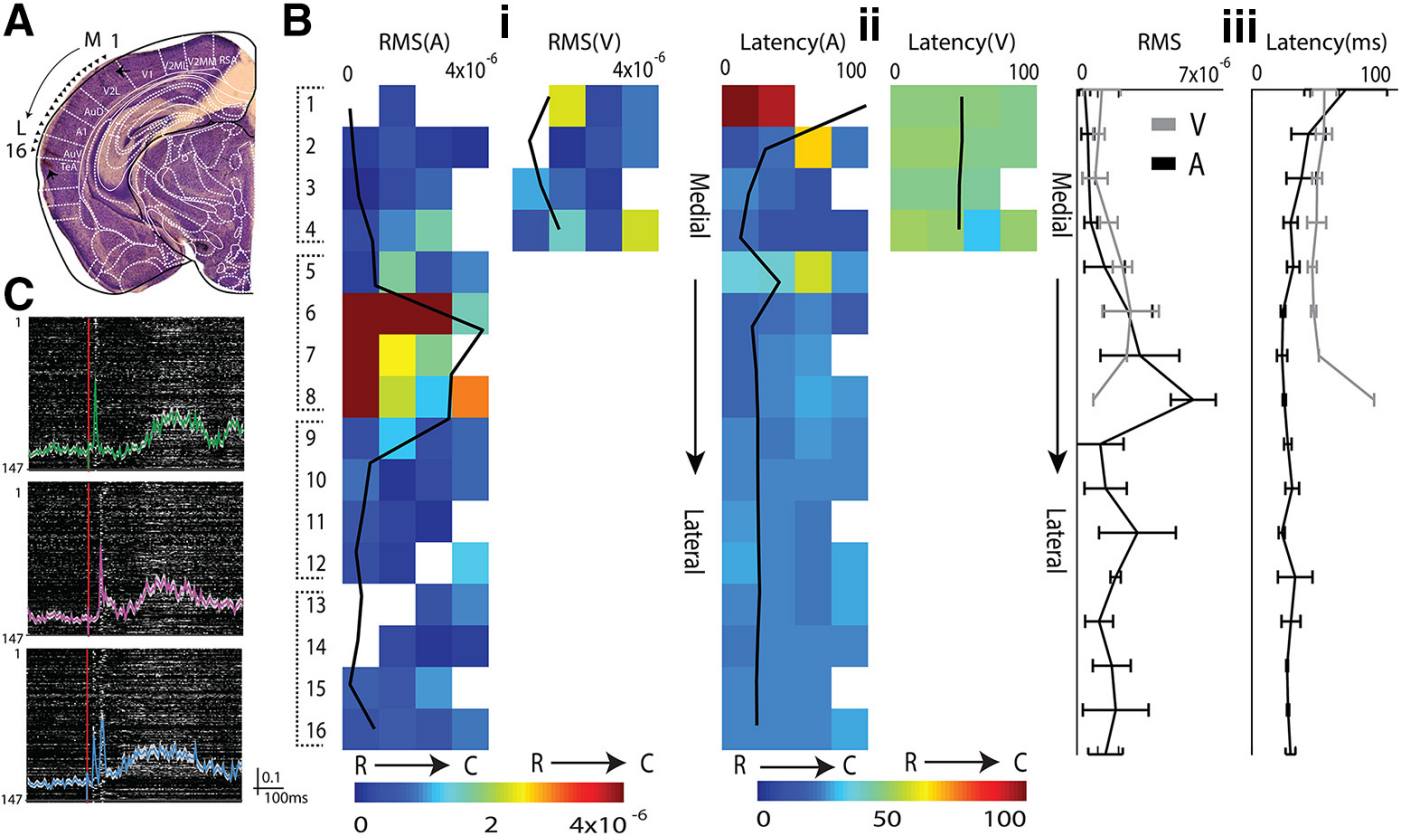
Origin of OFC afferents in the early sensory cortex, carrying auditory, visual, and multisensory information. ***A***, Nissil stain of an example brain section including the ACX showing electrode tracks (arrows) for recording locations. ***Bi***, The rms amplitude of LFP responses to *A* (left) and *V* (right) for each of the four successive (medial to lateral, ML) recording sites (as in ***A***) with 4 × 4 MEAs in an example mouse, overlaid with mean rms amplitude profile. ***Bii***, Latency profile of the locations in ***Bi*** for both auditory (left), visual (right), overlaid with mean latencies. ***Biii***, Mean ML profiles of rms response (left) and latency (right) to *A* and *V* averaged across four mice. ***C***, Normalized PSTHs of 147 multisensory single units recorded from regions of ACX and VCX immediately dorsal to A1 (*n *=* *18 mice) shown as rows in grayscale, in response to *A*, *V*, and *M* (upper, middle, and bottom). Mean of the normalized PSTHs with SEM (gray shading) in each case is overlaid. Vertical bar alongside is the length of normalized rate responses (a.u.).

In order to look at the possible sources of auditory, visual, and multisensory inputs into OFC, we injected fluorescent retrograde tracers into OFC (see Materials and Methods; Fig. 4, top row, *A*). The cortical region on the rostrocaudal axis encompassing the ACX (Fig. 4*B*), based on anatomic landmarks (Paxinos and Franklin, 2013), was imaged. Numbers of fluorescently labeled cell bodies (Fig. 4*C*,*D*; *n *=* *5 animals; see Materials and Methods) in different regions along the mediolateral extent up to the rhinal fissure (Fig. 4*B*, arrow) were determined. Regions were demarcated based on the mouse brain atlas (Paxinos and Franklin, 2013) and included the ACX: ventral, primary, and dorsal; and the visual cortex (VCX): lateral, primary, and medial (M/L) divisions. All regions have direct projections to the OFC, with secondary regions showing much stronger labeling than primary regions in both auditory and visual cortices (Fig. 4*D*). Thus, direct anatomic pathways carrying auditory and visual information to the OFC exist (Zingg et al., 2014) and could provide the necessary source of inputs giving rise to both unisensory and multisensory responses in the OFC. Other possibilities also exist, as observed by the retrograde labeling of regions such as TeA, Ect, Prh, PPC, basolateral nucleus of amygdala, known to be multisensory (Ghazanfar and Schroeder, 2006; Lyamzin and Benucci, 2019; Morrow et al., 2019). Among the auditory and visual sensory cortical regions projecting directly to the OFC, with LFP recordings along the mediolateral axis (Fig. 5*A*, electrode tracks, black arrows), we find that the dorsal ACX and lateral secondary VCX (AuD and V2L) have responses to both auditory and visual (Fig. 5*Bi*) stimuli, with expected latencies (Fig. 5*Bii*) in primary and secondary regions. Thus, the above secondary regions, with their strong projections to the OFC, are the most likely origins of multisensory (*A*/*V*) inputs to the OFC. Single-unit recordings in the AuD and V2L show multisensory responses in spike rates (Fig. 5*C*), as observed in the OFC (Fig. 1). Similar fractions of modulatory effects were found in the AuD as in the OFC [86/147, 58.5% *M0* (*z* = 0.67, *p* = 0.49), 23/147, 15.6% *ME* (*z* = −0.49, *p* = 0.62), and 38/147, 25.85% *MS* (*z* = 0.3, *p* = 0.76), two-proportion z test], showing that such modulatory effects are general in the multisensory responding regions. Further, these regions along with primary regions with direct projections or indirectly could also provide a separate only auditory and only visual input source to the OFC.

### Oddball stimulation to parse out modality-specific and multisensory synapses

**Figure 6.**
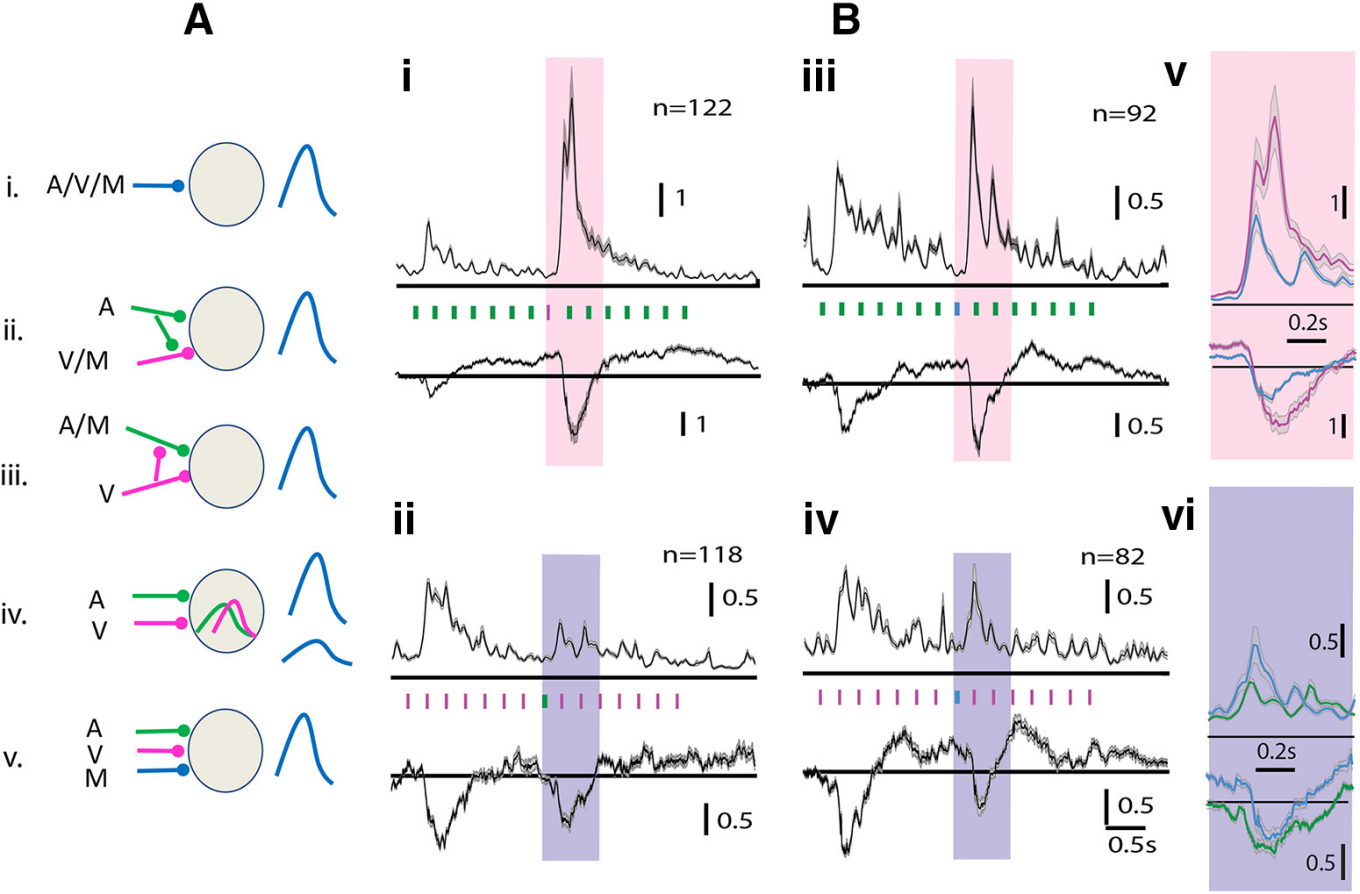
Multisensory oddball stimulation shows the presence of separate *A*, *V*, and *M* inputs to the OFC. ***Ai–v***, Possible models of synaptic inputs on a single OFC neuron. ***Bi–vi***, Multisensory oddball responses in mean normalized population PSTHs (and LFPs, below) are shown with (***i***) standard = *A* and deviant = *V*, (***ii***) standard = *V* and deviant = *A*, (***iii***) standard = *A* and deviant = *M*, and (***iv***) standard = *V* and deviant = *M*. Vertical bars between a PSTH and LFP (***Bi–v***) shows time of stimulus tokens. Colored rectangles (pink and purple) mark the response to the deviant, being compared (enlarged) in ***Bv*** and ***Bvi***, respectively.

With clear anatomic connections to the OFC from specific sensory regions, which may underlie the origins of separate *A*, *V*, and *M* inputs to the OFC including the possibilities shown in Figure 6*A*. We designed stimuli to rule out the cases, from a single unit and LFP responses of OFC. We used an oddball stimulation paradigm with multiple modalities, which makes use of the property of adaptation of synapses with repeated presentations of the same stimulus (called the standard; Fig. 6*Bi–iv*), eliminating the response of the postsynaptic neuron (in this case, OFC neurons), while allowing the postsynaptic neuron to still respond to another stimulus (deviant) that provides inputs through other un-adapted parallel synapses. Complete adaptation to one stimulus thus allows oddball stimulation to look for the presence of different kinds of synapses or input pathways. We used different modality or *M* stimuli in the oddball paradigm to investigate the presence of separate *A*, *V*, and *M* channels of input to the OFC single neurons. In all cases (Fig. 6*B*), we consider the normalized PSTHs (normalized by the mean response of first 0.5 s after stimulus start) of a population of single units. First, repeated presentation of 15 *A*, *V*, or *M* tokens at 4 Hz shows that neurons in the OFC get completely adapted to each of these stimuli by 1.7 s (decay time constants: *A*: 560 ms, *V*: 740 ms, and *M*: 80 ms), evident from the rates reaching spontaneous activity levels from the strong onset response. Thus, OFC neurons are completely adapted to the standard stimulus by the end of the seventh token, in such trains of standards (4 Hz).

We use the eighth position, 1.75 s from stimulus stream onset, in trains of tokens for the *D* oddball stimulus. In the case of unisensory standard and deviant, OFC neurons responded to the onset/first of the standard stimulus tokens (*A* or *V*) and then again to the deviant stimulus (*V*/*A*; Fig. 6*Bi*,*ii*). The above results suggest that there are parallel input pathways to OFC that are capable of carrying only *A* and only *V* inputs, thus ruling out the possibility in Figure 6*Ai*, where the same pathway is carrying *A*, *V*, and *M* input. The inputs are not necessarily unisensory, but could also be *M* in nature, one responding to *M* and *A* but not *V* with a separate *V* only input (Fig. 6*Aiii*) and the other responding to *M* and *V* but not *A* with a separate *A* only input (Fig. 6*Aii*). So to further understand the nature of inputs we used *M* as the deviant token in streams of *A* or *V* as the *S* tokens (Fig. 6*Biii*,*iv*). If there are inputs that are of the kind which respond to either one of the unisensory stimuli and *M* than in the case of *M* as deviant in a stream of *A* or *V* there should be no response to the deviant. In both cases, the *M* stimulus as deviant evokes a response, indicating that there are exclusively unisensory inputs to the OFC, thus ruling out possibilities *Aii* and *Aiii* in Figure 6*A*.

Next, we consider whether there are separate *M* inputs to OFC, that is inputs that are active only when *M* stimuli are presented and not when unisensory (*A* or *V*) stimuli are presented. We compare the response to *M* as the deviant token, with the response to *V* (or *A*) as the deviant token in a stream of *A* (or *V*) as standard tokens. This difference in normalized spike rates [7.04 ± 11.86 and 2.69 ± 3.82, *p* < 0.001 (Fig. 6*Bi*,*iii*,*v*) and 1.13 ± 0.70 and 1.85 ± 2.74, *p* < 0.01 (Fig. 6*Bii*,*iv*,*vi*)] that occur between multisensory deviant and unisensory deviant in the presentation of auditory or visual standards, respectively (Fig. 6*Bv*,*vi*), indicates the presence of a separate multisensory synapse, ruling out possibility *Aiv* in Figure 6*A*. Similarly, the comparison in normalized LFPs obtained in each case and their difference [1.49 (Interquartile range, IQR = 2.39), 0.77 (IQR = 0.98) *p* < 0.001 (Fig. 6*Bi*,*iii*,*v*) and 0.45 (IQR = 0.98), 0.31 (IQR = 0.50), *p* < 0.05 (Fig. 6*Bii*,*iv*,*vi*), median compared due to outliers] further indicates that such parallel paths are not emergent in the OFC.

### Rapid differential plasticity with audiovisual pairing in the OFC

We find that congruent audiovisual stimulus presentation shows different types of modulatory effects over the single neuron unisensory responses in the OFC. Further, we show that the three stimuli *A*, *V*, and *M* are represented differently with unique response properties based on rates and basic temporal response characteristics. The three stimuli can be decoded or discriminated from each other based on various response temporal features. In order to investigate the effect of continuous exposure to multisensory stimuli, we considered the effect of repeated pairing of *A* and *V* in responses of single neurons to the component *A* and *V* stimuli subsequently to understand how the separate input pathways (deciphered from oddball experiments) interact to change representation of the component stimuli.

**Figure 7.**
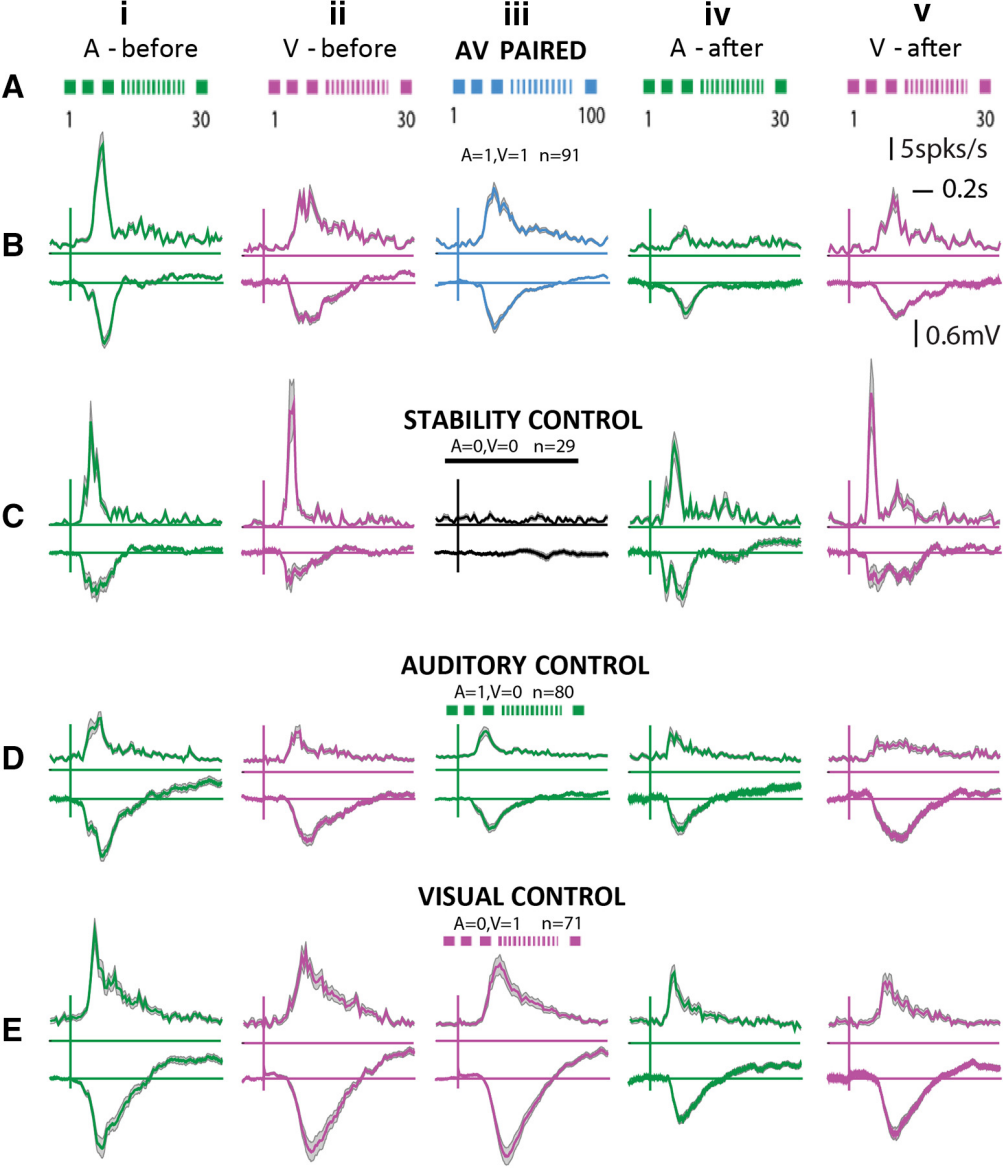
Population effects of audiovisual associations in the OFC. Row ***A***, Experimental protocol is displayed in cartoon form along with reponse PSTHs for the same conditions. ***B–E***, Each row shows PSTHs with SEM of populations of OFC single neurons combined across different experiments. Row ***B***, Case of audiovisual pairing. Row ***C***, Stability control (no exposure in between). Row ***D***, Auditory only control. Row ***E***, Visual only control. First two columns of all rows show mean responses (to 30 iterations) to *A* and *V* before the A-V pairing (or each of the control exposure), and the last two columns of all rows show the same after A-V pairing (or each of the control exposure). The middle column shows mean responses (to 100 iterations) during the A-V pairing (or each control exposure).

Following presentations of *A* and *V* stimuli separately (30 repetitions each; Fig. 7*Ai*,*ii*,*Bi*,*ii*) to characterize the basic response to unisensory components, 100 repetitions (one pairing every >7 s) of congruent *A* and *V* were presented (Fig. 7*Aiii*,*Biii*). Following the pairing responses to *A* and *V* (Fig. 7*Aiv*,*v*,*Biv*,*v*) were again collected to be compared with responses pre-pairing to identify the effects of rapid plasticity, if any. Counterbalancing the *A* and *V* before and after pairing did not alter the results. As controls, to rule out the effects of the elapsed time and habituation to the presentations of each of the unisensory components, during pairing, separate experiments were done (Fig. 7*C–E*). For the control experiments, instead of pairings, only the auditory stimulus (*A *=* *1, *V *=* *0; Fig. 7*D*) was presented in one case, only the visual stimulus (*A *=* *0, *V *=* *1; Fig. 7*E*) in another, and no stimulus (*A* = 0, *V* = 0; Fig. 7*C*) where time equivalent to 100 pairings was given, and the spontaneous activity was recorded. In each of the control experiments, at the end of the control exposures, responses to *A* and *V* were collected again (Fig. 7*C–Eiv*,*v*).

**Figure 8.**
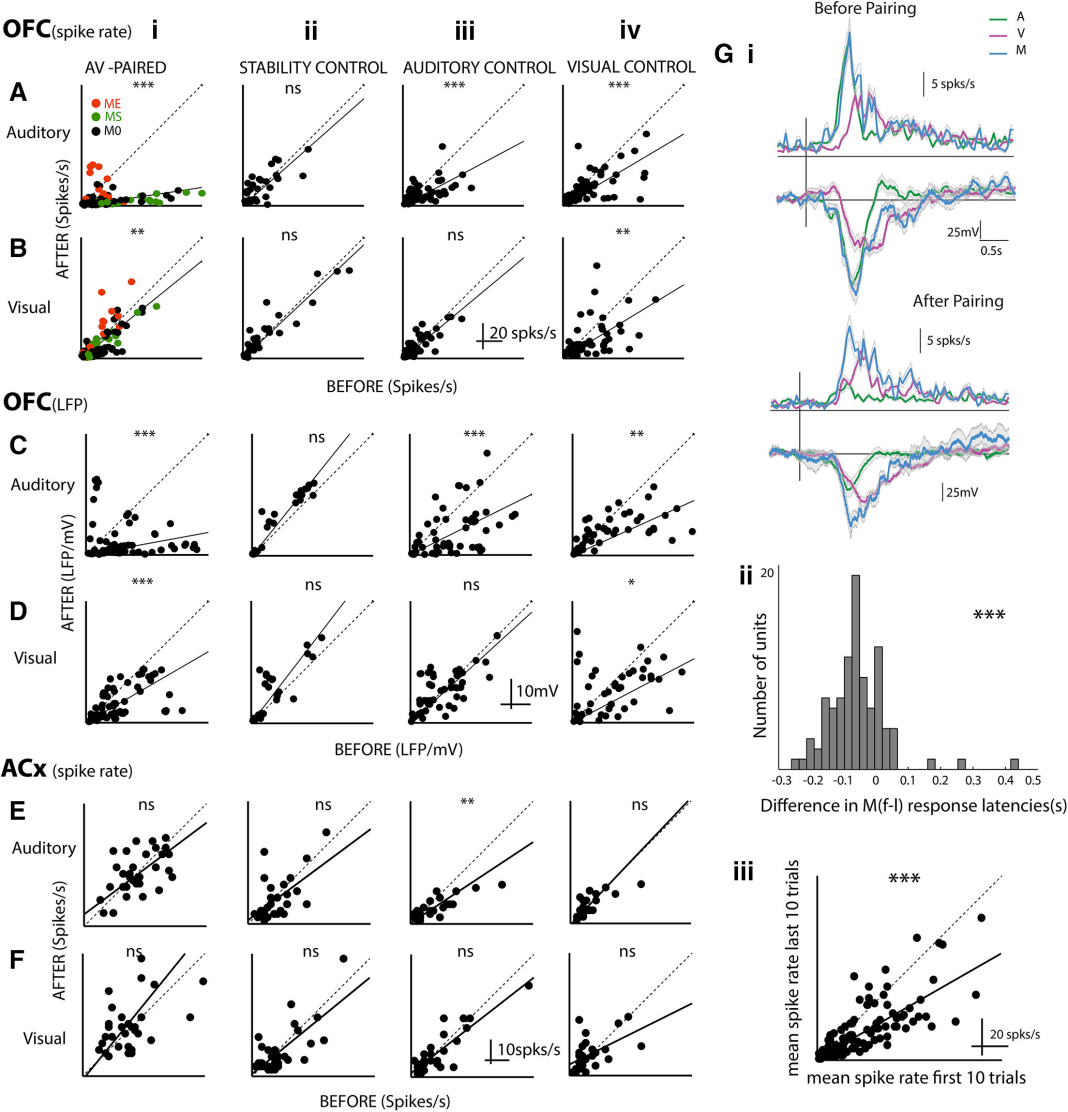
Differential rapid plasticity in *A* and *V* responses with *A-V* association. ***A–F***, Each panel is organized in the same way showing scatter plots of after versus before responses of single neurons in OFC-spikes (***A***, ***B***), OFC-LFP (***C***, ***D***), and ACX-spikes (***E***, ***F***) to auditory (***A***, ***C***, ***E***) and visual (***B***, ***D***, ***F***) stimuli. ***A–D*** are for data from the OFC, and ***E***, ***F*** are for data recorded in AuD/V2L. Each row has four plots (***i–iv***) starting with *A-V* pairing followed by the three control cases. ***Gi***, The response to *A* and *V* before (upper) and after (lower) pairing is shown with the population mean PSTHs (above) and mean LFPs (below). Overlaid on them are responses to *M* (before: based on the first 10 trials of pairing and after: based on the last 10 trials of pairing). ***Gii*,*iii***, Distribution of latency differences between first and last 10 trials of pairing response and scatter plot of mean response rate of last 10 trials versus the first 10 trials. *p < 0.05, **p < 0.01, ***p < 0.001, ns = non significant.

In the population of OFC neurons, we observed rapid plastic changes in responses to *A* and *V* due to passive exposure to repeated congruent *A*-*V* stimulation. Following the pairing the spike rate of OFC single neurons in response to both *A* (paired *t* test, *t*(df = 90) = 7.48, *p* < 10^−11^; Figs. *7Biv*, *8Ai*) and *V* (paired *t* test, *t*(df = 90) = 3.27, *p* = 0.001; Figs. 7*Bv*, *8Bi*) were suppressed. The suppression of responses to *A* was stronger compared with the mild suppression seen in *V* responses (one-sided comparison of slopes based on bootstrap confidence intervals; Materials and Methods; slopes *A*: 0.1521 and *V*: 0.8071). Significant suppression was also observed in the LFPs following pairing but to different degrees (paired *t* test, *t*(df = 80) = 5.99, *p* = 10^−8^ for *A*, slope 0.1885, paired *t* test, *t*(df = 80) = 4.98, *p* = 10^−6^ for *V*, slope 0.5892; Fig. 8*C*,*Di*). Strength of the depression of *A* responses in LFPs was the same as that in spike rates (slopes for before after spike rates 0.1521 and before after LFP rms 0.1885, NS, non-significant, bootstrap analysis; Materials and Methods), indicating that the long-term depression observed in the OFC auditory response spiking activity is derived from the input pathway. However, there was large variability in the before and after LFP relationship with some LFP channels showing no change or enhancement. The suppression to *V* after pairing was stronger in LFPs than in spike rates (one-sided comparison with bootstraps; Materials and Methods) indicating that there is a net enhancement on top of the already suppressed visual activity input to the OFC.

The *ME*, *MS*, and *M0* units (Fig. 8*Ai*,*Bi*, different colors) showed distinct effects both in auditory and visual responses. The multisensory modulation that was observed, with the audiovisual association changed the responses in a manner so as to make the effect of modulation stronger and longer term. The *ME* units showed relative enhancement of *A* responses compared with the population effect on *A* responses of pairing (*ME* after vs before slope 0.6246 significantly greater than 0.1521; Materials and Methods). The effect on *M0* units was not significantly different from the population (*M0* slopes 0.1647), while the *MS* units were significantly more suppressed than the population (*MS* slope 0.1000). Thus, if the overall effect of pairing is considered to occur in all units, then the final auditory responses after pairing were determined by the kind of multisensory modulation that operated on the overall effect. A similar effect was seen on visual responses as well following audiovisual pairing. Although *M0* and *MS* units did not show any significant difference in their after versus before relations due reflected in the slopes (*MS* slope 0.7087, *M0* slope 0.6741, NS; Materials and Methods), the *ME* units showed a strong enhancement of visual responses (*ME* slope 1.4045, significantly greater than overall slope 0.8071; Materials and Methods).

To rule out a general reduction in spike rates over time, control experiments were performed and comparisons were made between responses before and after a time period equivalent to the duration of pairing with no stimulus presentation (*A *=* *0, *V *=* *0; Figs. 7*C*, 8*Aii*,*Bii*). Neither rate responses to *A* nor that to *V* changed between before and after the no stimulus period (Figs. 7*C*, 8*Aii*,*Bii*). The same was observed in LFPs (Fig. 8*Cii*,*Dii*). Thus, the recordings are stable throughout the duration of the experiments and hence the effects seen cannot be due to fluctuations that can occur over time.

To further rule out habituation in auditory responses (or visual responses; Taaseh et al., 2011) to the repeated presentation of *A* (or *V*), control experiments with 100 presentations of only *A* (*A* = 1, *V* = 0, or only *V*, *A* = 0, *V* = 1), instead of pairing, were performed. Habituation-based reduction of responses to *A* was observed on repeated *A* presentations (paired *t* test, *t*(df = 79) = 4.27, *p* < 10^−5^; Figs. 7*Div*, 8*Aiii*), and the suppression was weaker than that observed in pairing (before after slopes in case of *A1V1*: 0.1521 and in case of *A1V0*: 0.5370, one-sided comparison with bootstraps; Materials and Methods). Thus, the repeated presentation of *A* could not explain the long-term depression in *A* responses with pairing. Further, the observed suppression in LFPs and that in spike rates in response to *A* after *A *=* *1, *V *=* *0 exposure had no difference (bootstrap analysis, NS), indicating that the habituation observed with only *A* exposure is due to the habituation in the input pathway. Visual responses, both in spike rates and in LFPs, did not change after *A *=* *1, *V *=* *0 exposure compared with that before (spike rates: paired *t* test, *t*(df = 79) = 1.25, *p* = 0.21, LFP: paired *t* test, *t*(df = 55) = −0.41, *p* = 0.67). The *A *=* *0, *V *=* *1 exposure produced suppression in LFPs between after and before responses to both *A* and *V* (*A*: paired *t* test, *t*(df = 58) = 4.47, *p* = 10^−5^, *V*: paired *t* test, *t*(df = 58) = 2.54, *p* = 0.01; Fig. 8*Civ*,*Div*). Similarly, in spike rate responses also, suppression was observed to both *A* and *V* (Fig. 8*Aiv*,*Biv*). The suppression in spike rates in response to *V* after *A *=* *0, *V *=* *1 is stronger than that after pairing *A *=* *1, *V *=* *1 [one-sided comparison of slopes based on bootstrap confidence intervals (Materials and Methods), slopes 0.6116 for *A0V1* and 0.8071 for *A1V1*], also further indicating that pairing effectively induces an enhancement in visual responses in the OFC.

Thus, from the above experiments, we find that all exposures lead to suppression in the *A* responses, and after pairing, the suppression is stronger than that after only *A* and only *V* exposures, and also stronger than the net multiplicative effect of the individual suppressions (multiplicative slope for *A*, *A1V0xA0V1* 0.3200 higher than *A1V1* slope 0.1521, bootstrap comparison; Materials and Methods). On the other hand, the net multiplicative effect on visual responses (multiplicative slope for *V*, *A1V0xA0V1* 1 × 0.6116) was lower than the slope observed in the case of pairing (*A1V1* slope for *V* 0.8071, bootstrap comparison; Materials and Methods). Also, pairing causes suppression in *V* responses in LFPs and hence a net enhancement of the *V* responses over the inputs is observed in the spike rate of OFC neurons. Additionally, with pairing, the suppression observed with only visual exposure is overcome. Thus, overall, we find a differential rapid plasticity of *A* and *V* responses with multisensory pairing. Considering the responses in the last 10 trials of the pairing as the final representation of the *M* stimulus following pairing, we find that the representation of *M* is close to only a visual response showing the visual bias in representation of *M* (Fig. 8*Gi*). Similarly, comparing the latency difference in the first 10 trials and last 10 trials of pairing shows an increase in latency (Fig. 8*Gii*), median 60 ms, same as the difference in latency of *M* and *V* (Fig. 3*Aiv*), also showing the *M* response following pairing to be more like the *V* only response. An effective decrease in overall response is observed (Fig. 8*Giii*) between the first 10 and last 10 trials of the pairing also consistent with the idea of the *M* response being dominated by the *V* component.

To further understand the effects of pairing, we considered the same experiments to look at effects of the pairing and the related control exposures on *A* and *V* responses in single neurons of AuD/V2L, one of the likely origins of the multisensory inputs (Fig. 4) to the OFC. The same exposure experiments and quantification were performed as done in the OFC (Figs. 7*A*, 8*A–D*), and the data are summarized in Figure 8*E*,*F*. No changes in spike rate responses to *A* or *V* were observed before and after any of the exposures except a suppression in *A* responses after *A *=* *1, *V *=* *0 exposure (paired *t* test, *t*(df = 30) = 3.03, *p* = 0.005). Thus, effectively a mild enhancement in *A* responses can be concluded in AuD/V2L with the pairing. Thus, the origins of the multisensory inputs to the OFC do not show any of the effects observed in the OFC with pairing, and the changes are derived from higher-order regions. Thus, although similar types and same relative abundance of multisensory modulation was observed in the AuD/V2L and OFC, the effects of pairing to create associations between the *A* and *V* stimuli showed primary effects in higher-order regions than AuD/V2L, including the OFC.

### Proposed network model

To explain the difference in latency between the *A* and *M* stimulus, we hypothesize that neurons in the OFC receive inputs from a separate pathway activated only by multisensory inputs but not by the individual unisensory components. This multisensory pathway also likely inhibits the auditory inputs to the OFC for achieving lower latency to auditory stimulation. For the above hypothesized model to achieve the latency differences, a “low threshold multisensory-activated inhibition” on the auditory inputs is required. The suppression by the multisensory stimuli in units responding strongly to auditory provides further evidence in favor of the existence of such an inhibitory input. The same could also be achieved by a pathway that is activated by both an “only visual” and “a multisensory” stimulus. However, the latter is less likely due to the very large difference (∼100 ms) in latency between the auditory and visual responses of OFC single units.

From above, it is likely that there are three separate auditory, visual, and multisenory inputs on to a single OFC neuron. This possibility is further consolidated from oddball stimulation experiments where we show the presence of distinct auditory, visual, and multisensory inputs by using stimulus-specific adaptation. In the case of *A* standards, the response to the *V* deviant is larger than the *M* deviant (Fig. 6*Bv*), while in case of *V* standards, the response to the *M* deviant is stronger than the *A* deviant (Fig. 6*Bvi*).This differential effect suggests that there is a “multisensory inhibitory” input as well to cause the decrease in response to deviant *M* compared with deviant *V* in a stream of *A* standards. A comparison of LFPs of the same also suggests that the multisensory inhibition may be driven from outside OFC. Thus, as observed, based on latencies (Fig. 3*A*) and with the oddball experiments, we conclude that other than separate excitation pathways both *V* and *M* also provide inhibition to the OFC. The only visual exposure from the control condition of our pairing experiment in Figures 7, 8 causing the *A* responses to be suppressed (Fig. 8*Aiv*) with a similar change in LFPs shows that repeated multisensory and visual presentation strengthens the inhibition of the *A* responses by *M* and *V* in the inputs to OFC. Hence the inhibition of *A* by *V* and *M* as hypothesized earlier based on latencies and suppressive multisensory modulation is further corroborated.

**Figure 9.**
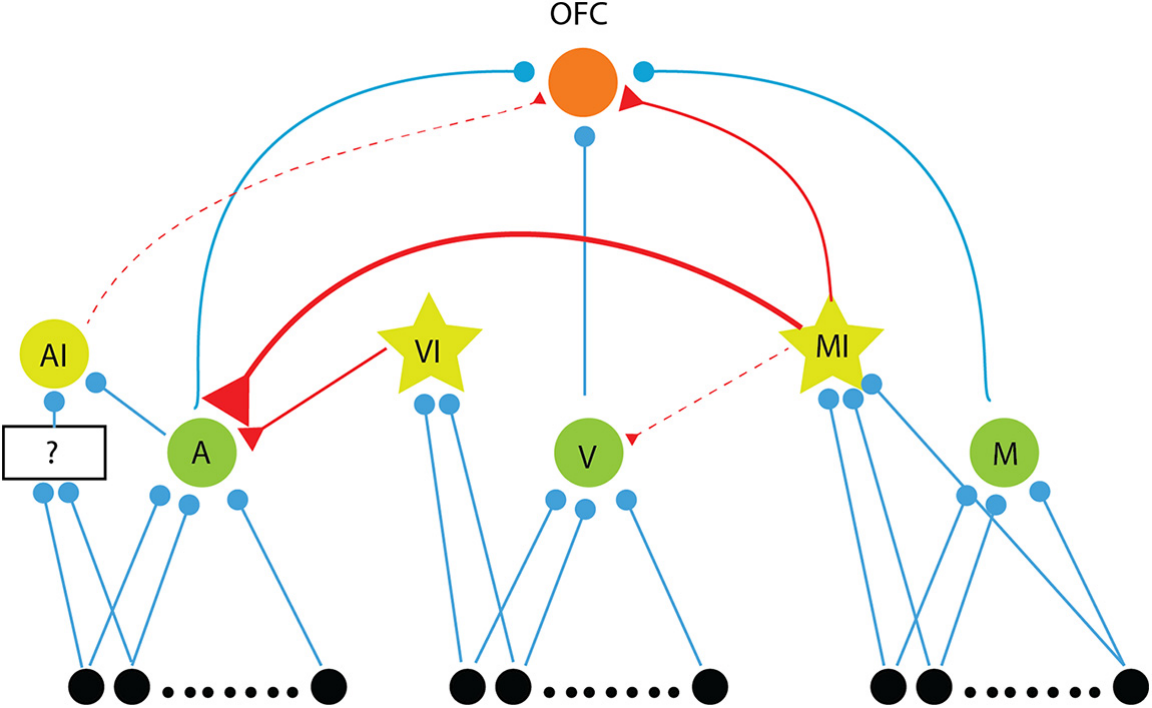
Network model to explain the observations. Hypothetical network model showing inputs to the OFC: three excitatory inputs, *A*, *V*, and *M* (green neurons, blue synapses) and two inhibitory inputs, *AI* and *MI* (yellow) neurons with red synapses. *MI* (a low threshold inhibitory) also inhibits the *A* and *V* input neurons and another inhibitory neuron *VI* (low threshold inhibitory) inhibits *A*. Three separate input populations, along with a parallel (unknown, indicated by black box) auditory input to OFC (black neurons projecting to *A*, *V*, and *M*) activated in the presence of *A*, *V*, and *M* stimuli, respectively (*M* stimulus also activates inputs to *A* and *V*) form the overall inputs to the network.

Based on our results, we propose a hypothesized minimal network model that qualitatively explains our observations (Fig. 9). We consider the three excitatory input neurons (*A*, *V*, and *M*) to the OFC, arising from three separate populations. Three inhibitory neurons are also considered, *AI*, *VI*, and *MI* (from our discussion above), with *AI* receiving parallel auditory inputs. The inputs to *M* are activated by an *M* stimulus only and not activated with only *A* stimulus or only *V* stimulus. The *A* and *V* input populations are both activated along with the *M* input population for the *M* stimulus. The *A* neurons providing input to the OFC receive inhibitory inputs from both the inhibitory neurons. The *AI* neuron provides a weak inhibitory input to the O neuron. The *MI* neurons inhibit *A* neurons strongly and the *V* neurons weakly and also provide inhibitory input to the O neuron. The *MI* and *VI* neurons are lower threshold than *V* and *M* neurons to inhibit *A* with *M* stimulation. The synapses are considered to be depressive in nature (Mill et al., 2011), which is also the basis for our multisensory oddball paradigm results. With the above, a latency difference as observed can be explained, which allows the O neuron to be responding with the latency determined by the *M* neuron for *M* stimulus with *A* getting inhibited. With the *AI* neuron inhibiting the O neuron in a delayed manner would allow for the response durations for *A* to be shorter than that for *V* and *M*. Comparatively, in case of the *M* stimulus, the *MI* and *AI* inhibition on O can be overcome by the additional *A* and *V* inputs in case of *M* unlike in case of *A*, above. Thus, *M* can have longer response duration. When there is an imbalance between auditory and visual excitatory drives, specifically when the auditory drive is stronger than the visual drive, OFC neuron faces stronger inhibition from *AI*, causing suppression of responses to multisensory stimulus. This may not happen if the visual drive is equivalent to auditory drive, along with parallel excitation from multisensory pathway, providing enough excitatory input for OFC neuron to show enhancement of response to multisensory stimulus. With depressing excitatory synapses, the SD stimulus responses can also be explained. Particularly, the decrease in *M* response compared with *V* response as deviants in streams of *A* standards is explained by the *MI* input to O. For the rapid plasticity observed, with repeated AV pairing, O neurons respond strongly to *M* inputs inhibiting *A*, and thus depression in the *A* -> O synapse. As the inhibition from *M* to *V* is weak and *V* still responds during *M* stimulus, causes enhancement of the *V* -> O synapse. The above would also lead to the strengthening of the *M* -> O synapse.

## Discussion

While the OFC has been extensively studied in the context of behavior, little is known about the basic response properties of OFC neurons to sensory stimuli, especially in the mouse. In this study, we consider single neuron responses of OFC to auditory and visual stimuli and also to audiovisual (multisensory) stimuli. Our study shows that the multisensory nature of the mouse OFC is in concurrence with other similar studies in the Ventrolateral PFC of primates (Rolls, 2004; Sugihara et al., 2006; Jung et al., 2018). Comparing responses to component unisensory stimuli and response to the multisensory stimulus (Miller et al., 2015), we find that about equal proportions of neurons to be modulated and otherwise with simultaneous presentation of the unisensory stimuli. The lack of spike rate change in 54% neurons from the maximum unisensory response in case of the multisensory stimulus suggests no multisensory modulation but is, in fact, a nonlinear interaction. The other half of neurons showed either enhancement or suppression depending on the strength of responses to the auditory component, showing that auditory inputs guide initial modulation by *M* stimuli. The neurons also were found to encode stimulus identity in specific temporal profiles which are broadly reflected in the latencies and duration of response to different stimuli. The presence of nonlinear interactive effects for the multisensory stimulus even with a 100-ms difference between responses to the auditory and visual components shows likely subthreshold mechanisms involved in causing the observed multisensory modulations.

Latency to the three stimuli in this study is in concurrence with previous literature from Ventrolateral Prefrontal Cortex (Romanski and Hwang, 2012). We believe that for audiovisual integration to take place, the generation of a strong prediction in OFC about the late-arriving stimulus elicited by the first-arriving stimulus can cause such latencies to appear (Mazzucato et al., 2017). However, Rowland et al. (2007) suggest multisensory stimulus consisting of simple LED flash and noise results in shortest latencies in the superior colliculus of the cat, which could be the result of the subthreshold addition of modality-specific inputs crossing threshold sooner than either of the unisensory stimulation. Hence, the difference may lie in distinct subthreshold properties and receptive fields of the neurons in the two regions.

We show that OFC neurons receive sensory inputs directly from the auditory and VCX, more from nonprimary than primary regions, and also possibly indirectly through TeA, Prh, PPC, and BLA, which are involved in processing temporal and spatial aspects of visual information and content of auditory stimuli. Thus, OFC has access to both filtered information from auditory-specific and visual-specific areas and other aspects of sensory-driven information from higher association areas, representing a redundant multilevel process of integrating information across different sensory systems. Our conclusion of the presence of separate auditory, visual, and multisensory inputs based on multisensory oddball experiments supports the above conclusion. Based on the *AV-*oddball stimulation results, we find there are effective parallel unisensory (*A* and *V*) and multisensory (*AV*) inputs to the OFC and the findings are summarized with additional components to explain our other results, in the proposed network model (Fig. 9). It is to be noted that we consider effective pathways, in the sense that there are likely multiple parallel *M* inputs and similarly *A* and *V* inputs. A further detailed network model would involve parsing out each one of them through pharmacological or optogenetic manipulations (Deisseroth et al., 2006). While multisensory audiovisual modulations are known to play a role in primary as well as secondary auditory and visual cortices (Bizley and King, 2008; Meredith et al., 2009; Hollensteiner et al., 2015) in sensory coding, as in our AuD/V2L responses, we find that similar modulations in similar proportions of neurons to also exist in the OFC. Thus, such multisensory modulations are likely intrinsic in the sensory pathways.

However, in terms of multisensory interactions, the most notable difference in multisensory processing observed between sensory cortices and the OFC is the outcome of multisensory associations or audiovisual pairing. We determined the effect of creating multisensory associations and their subsequent effect on unisensory stimulus processing in the OFC and AuD/V2L. We found differential plasticity in auditory and visual responses on long passive associations between the auditory and visual stimulus in the OFC. Such association driven plasticity was absent in the AuD/V2L single neurons. A visual bias was observed after multisensory associations with strong suppression of *A* responses and effective enhancement of *V* responses. Such differential rapid plasticity is intrinsic to the circuitry in OFC and independent of reward, behavioral context, or other cognitive aspects. The above result shows that the OFC circuitry intrinsically has a bias toward weighing visual stimuli differently from auditory stimuli in such associations when in behaving conditions the OFC would engage in multisensory tasks. Similar biases to olfactory or particular features of sensory stimuli have been observed (Kuang and Zhang, 2014); however, such sensory bias in associations absent in the sensory cortex and found in the higher cortex is not known. Further, since the OFC is also capable of modulating early sensory responses (Winkowski et al., 2013), such differential plasticity in the OFC can thus be critical in determining stimulus representation in the early sensory cortices in a multisensory environment.

Since the responses in the OFC are heavily dependent on behavioral state, based on attention, memory, hunger, or emotions, so to understand the intrinsic response properties to sensory stimuli in the OFC, we have considered the responses in an anesthetized preparation. Attention plays a major role in modulating responses in the frontal cortex and especially for visual stimuli It is known that eye position affects responses not only in VCX but also in the ACX in primates (Werner-Reiss et al., 2003), so potentially modulating responses in OFC as well. While we can ensure passive listening in awake state, eyeblink is not in our control as visual stimuli can also trigger eye blink which would interfere with the sensory responses in the OFC. So anesthetized recordings were done to eliminate the effect of all the above factors. This way we can gauge the intrinsic circuit’s characteristics independent of the state of the animal. Several other studies also make use of anesthetize preparation to eliminate the effect of brain state (Bizley and King, 2008; Atilgan et al., 2018). While anesthesia, in this case isoflurane, is known to alter responses through increased GABA_A_ receptor activation and NMDA receptor inhibition (Capey, 2007). In the OFC, we consider that the basic observations in our study would remain intact in terms of the properties of the circuits leading to sensory responses. Further, the results of rapid plasticity would also likely remain unchanged as NMDA receptor inhibition with isoflurane suggests stronger plastic changes to be expected without anesthesia.

Oddball stimulation has been used previously to study different frequency channels for probing frequency encoding in the ACX (Taaseh et al., 2011), and the effects can be explained by parallel channels of depressive synapses (Mill et al., 2011). In our study, we adapted it to study input channels from different sensory modalities, which in our case, very clearly reveals the presence of separate auditory, visual, and multisensory inputs into OFC. Based on the results of the oddball stimulation, we could also conclude the presence of an inhibitory input that is driven by multisensory stimuli to the OFC. It is to be further noted that recurrent connections, absent in our proposed hypothetical model (Fig. 9), can potentially generate and enhance the effects we have observed (Yarden and Nelken, 2017) in our oddball experiments. We have considered LFPs in parallel with spiking responses to loosely provide information on summed synaptic input activity and output activity respectively. Such comparisons as indicated also corroborate our conclusion of parallel input pathways for the three types of stimuli. If there are subthreshold depolarizations caused by inputs from synapses which we assume to be completely adapted with a sequence of standard presentations, then our conclusions need reconsideration. LFPs, known to indicate summed synaptic activity, however, do not show any evidence of synaptic inputs on presentations of repeated standards beyond the first two to three tokens.

Our finding of the OFC in the mouse being primarily multisensory (audiovisual) was not known before. The sensory inputs to the mouse OFC mostly studied have been unisensory in nature and primarily the chemical senses associated with food. Although responses in the mouse OFC to auditory stimuli (Winkowski et al., 2018) are known, there have been almost no previous studies of mouse OFC responses to visual stimuli or audiovisual stimuli. Thus, our findings are critically important for understanding how sensory responses are dynamically modified to achieve a stable perception of a dynamic environment and how changing value based on the behavioral relevance of incoming stimuli may be computed. Most importantly our findings indicate an intrinsic bias toward visual components over auditory components in the representation of multisensory stimuli in the OFC. Such a differential effect on one sense over the other would be critical in multisensory association-based behavior and value computation and similarly in adjusting sensory representations in a dynamic multisensory environment. Since the OFC is capable of modifying sensory responses (Winkowski et al., 2013), such a visual bias can cause behaviorally relevant visual components of multisensory stimuli to modify auditory responses during reward-based learning.

## Synthesis

Reviewing Editor: Christine Portfors, Washington State University Decisions are customarily a result of the Reviewing Editor and the peer reviewers coming together and discussing their recommendations until a consensus is reached. When revisions are invited, a fact-based synthesis statement explaining their decision and outlining what is needed to prepare a revision will be listed below. Note: If this manuscript was transferred from JNeurosci and a decision was made to accept the manuscript without peer review, a brief statement to this effect will instead be what is listed below.

The authors have adequately addressed the previous reviewers’ comments from the Journal of Neuroscience submission. The manuscript adds important information on multi sensory processing in a mouse model.

Minor comment:

Please review Figure 2 and make the colors in the figure and in the caption the same.
